# Supplementary material for: Prognostic Implications of Epilepsy Onset Age According to Relapse Pattern in Patients with Four-Year Remission
Source: Diagnostics (Basel). 2020 Dec 14;10(12):1089. doi: 10.3390/diagnostics10121089 (PMC7765101; doi:10.3390/diagnostics10121089)
Supplement: Supplementary file 1 [file diagnostics-10-01089-s001.pdf]

Table 1. Demographic data regarding the duration of remission according to relapse pattern in the age at onset before age 30.

**Age of onset group, n, (%)[a]**	**Under 10 years old, n = 43 (9.1)**				**10-19 years old, n = 192 (40.7)**				**20-29 years old, n = 85 (18.0)**			
**Relapse pattern**	**SF**	**LR**	**ER**	**P-value[b]**	**SF**	**LR**	**ER**	**P-value[b]**	**SF**	**LR**	**ER**	**P-value[b]**
n (%)	14 (32.6)	12 (27.9)	17 (39.5)		62 (32.3)	77 (40.1)	53 (27.6)		22 (25.9)	39 (45.9)	24 (28.2)	
Mean duration of the total seizure-free period ± SD, m	87.21 ± 34.98	74.25 ± 16.58	69.41 ± 15.87	N-C	76.35 ± 25.57	71.09 ± 24.00	70.30 ± 24.91	N-C	75.00 ± 26.23	69.77 ± 26.01	71.00 ± 20.81	N-C
Mean duration from last seizure to the initiation of AED withdrawal ± SD, m	72.79 ± 31.59	55.58 ± 15.14	55.94 ± 13.91	N-C	60.52 ± 22.10	56.10 ± 21.33	58.21 ± 22.35	N-C	59.05 ± 24.99	55.59 ± 20.47	55.63 ± 17.90	N-C
Mean duration of the AED withdrawal process ± SD, m	14.33 ± 8.07	18.67 ± 10.86	13.47 ± 6.92	N-C	15.84 ± 10.03	15.23 ± 8.35	12.09 ± 8.37	N-C	15.95 ± 9.44	15.46 ± 7.48	14.96 ± 11.78	N-C

SF, seizure-free; LR, late relapse; ER, early relapse; m, month; AED, antiepileptic drug. a: The proportion of all patients in each 10-year age group b: No significant differences in the mean duration among the subgroups stratified by relapse pattern across onset age groups.

Table 2. Demographic data of the duration to remission according to relapse pattern in the age at onset of 30 years old or older.

**9**	**30-39 years old, n = 53 (11.2)**				**40-49 years old, n = 55 (11.7)**				**50 years old or older, n = 44 (9.3)**			
**Relapse pattern**	**SF**	**LR**	**ER**	**P-value[b]**	**SF**	**LR**	**ER**	**P-value[b]**	**SF**	**LR**	**ER**	**P-value[b]**
n (%)	19 (35.8)	21 (39.6)	13 (24.5)		30 (54.5)	19 (34.5)	6 (10.9)		26 (59.3)	14 (33.3)	4 (7.4)	
Mean duration of the total seizure-free period ± SD, m	65.53 ± 12.53	74.95 ± 25.76	72.23 ± 23.46	N-C	80.73 ± 27.65	69.16 ± 15.46	74.50 ± 16.77	N-C	77.77 ± 24.00	74.14 ± 28.16	61.50 ± 8.74	N-C
Mean duration from last seizure to the initiation of AED withdrawal ± SD, m	49.05 ± 8.90	58.24 ± 23.55	56.23 ± 21.19	N-C	63.63 ± 26.87	53.00 ± 12.22	58.33 ± 19.81	N-C	57.88 ± 16.51	62.57 ± 28.35	51.50 ± 12.79	N-C
Mean duration of the AED withdrawal process ± SD, m	16.47 ± 7.76	16.71 ± 10.31	16.08 ± 11.35	N-C	17.77 ± 10.07	16.16 ± 6.06	16.17 ± 7.94	N-C	19.88 ± 11.81	11.57 ±5.80	9.50 ± 4.93	N-C

SF, seizure-free; LR, late relapse; ER, early relapse; m, month; AED, antiepileptic drug. a: The proportion of all patients in each 10-year age group. b: No significant differences in the mean duration among the subgroups stratified by relapse pattern across onset age groups.
